# Chaos syndrome

**DOI:** 10.1259/bjrcr.20160046

**Published:** 2017-03-14

**Authors:** Umesh Shriniwas Mudaliyar, Sneha Sreedhar

**Affiliations:** ^1^HOD, Department of Radiology, Bhaktivedanta Hospital and Research Institute, Mumbai, India; ^2^Pediatric Dentist, Department of Dentistry, Bhaktivedanta hospital and Research institute, Mumbai, India

## Abstract

Congenital high airway obstruction syndrome (CHAOS) is the obstruction of the foetal upper airways, which may be partial or complete. It is usually incompatible with life. Prenatal recognition of the disease is quite important. We report here a case of CHAOS diagnosed on antenatal ultrasonography.

## Introduction

Congenital high airway obstruction syndrome (CHAOS) is defined as complete or partial obstruction of the foetal upper airways. This clinical condition was brought into notice firstly by Hedrick in the late 1900s.^1^ CHAOS is usually caused by atresia or stenosis of the larynx or trachea. The true incidence of CHAOS is unknown. If the syndrome is unrecognized during the prenatal period, it usually results in stillbirth or death shortly after delivery.^2^ Fortunately, more cases can be recognized *in utero* nowadays, as there are significant technical improvements in prenatal imaging. Bilaterally enlarged hyperechoic lungs, dilated airways and flattened or inverted diaphragm are the typical prenatal sonographic findings. Foetal ascites and non-immune hydrops may also be associated with the clinical condition.^3^ Owing to the recently described management options, prenatal definition of foetal airway obstruction has come into prominence with the hope of neonatal outcome improvements.^4^ We present the case of foetus with such secondary changes diagnosed during routine ultrasound evaluation in 24 weeks gestation.

## Case report

A 27 year-old female came to the hospital for routine second trimester antenatal ultrasound. Clinical history did not reveal any predisposition to increased risk for genetic or familial disorder. Initial first trimester ultrasound scan was done at 6 weeks which did not show any significant abnormality. Then she reported directly at 24 weeks of gestation for follow-up second trimester foetal anomaly scan. Apart from second trimester ultrasound, other routine antenatal investigations did not reveal any significant abnormality. The foetal karyotype was normal.

**Figure 1.**
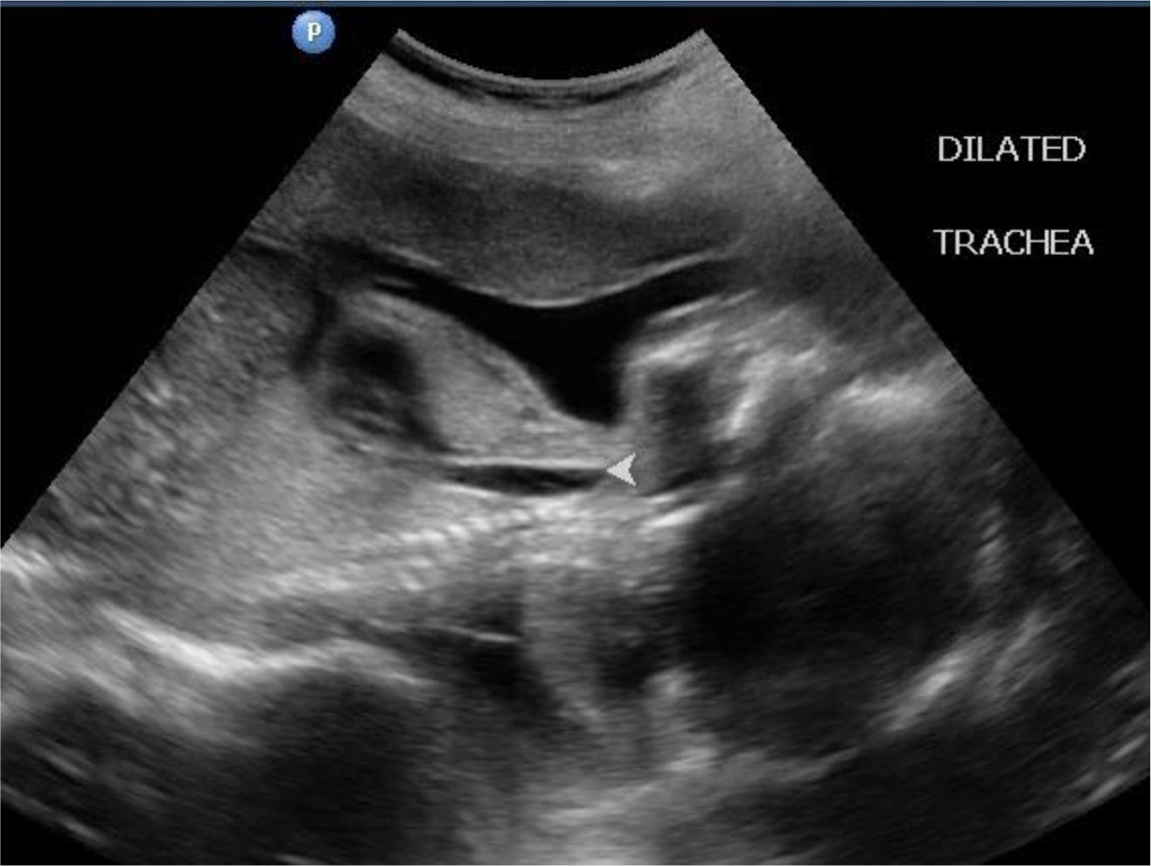
Dilated trachea/bronchi: distal to the obstruction seen on midsagittal view of chest.

**Figure 2.**
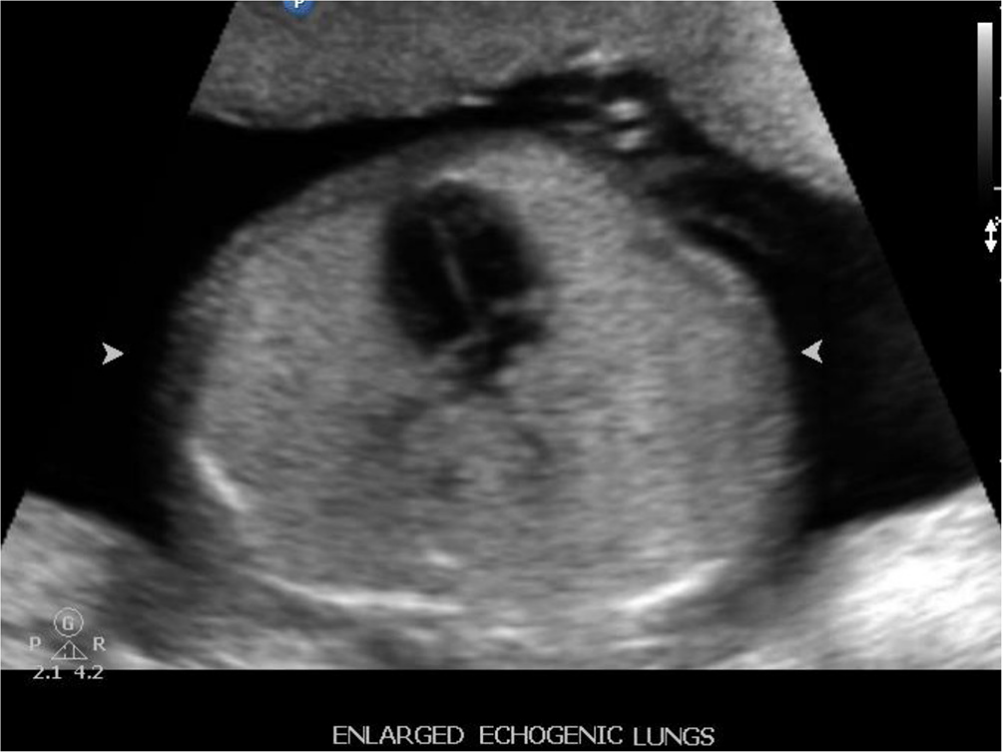
Enlarged and echogenic lungs seen on sectional image of chest.

**Figure 3.**
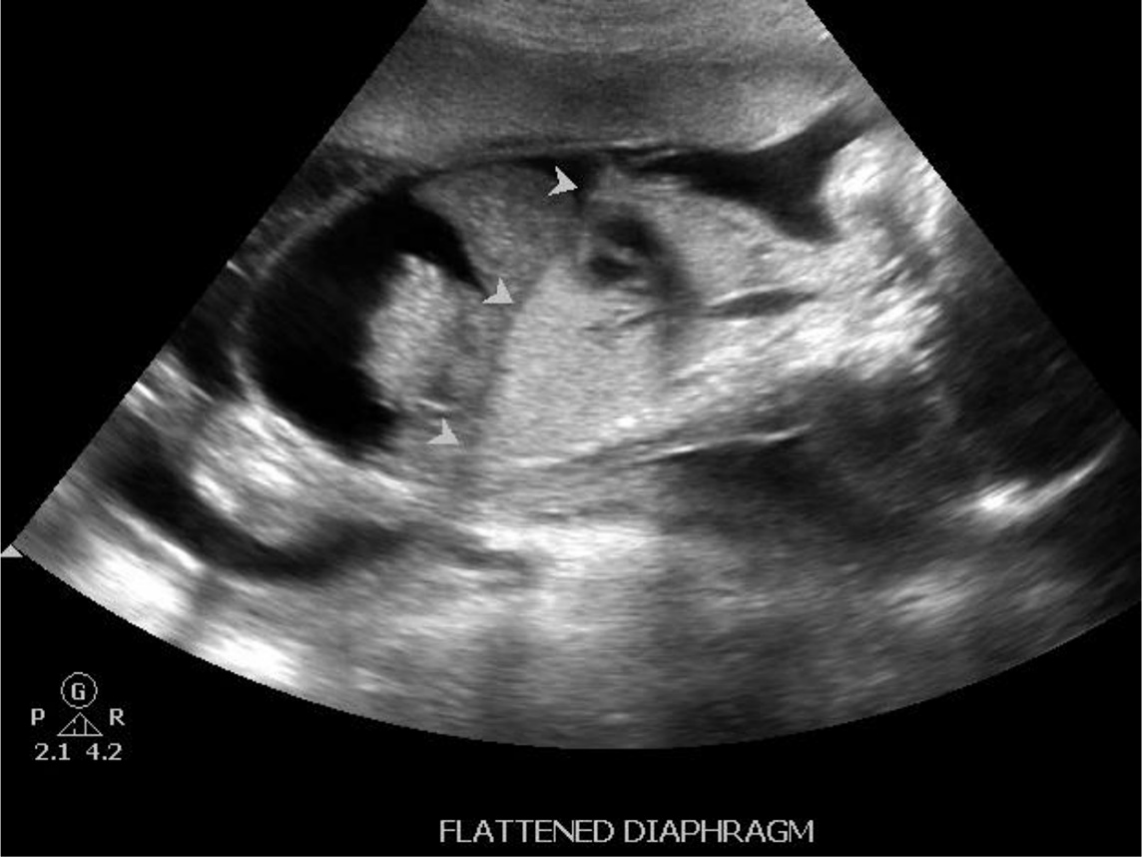
Diaphragmatic inversion seen on parasagittal view of thoracoabdominal region.

The classical ultrasound signs of **“CHAOS”** are as follows:

- Dilated airways below the level of obstruction (Figure 1).
- Hyperexpanded and hyperechoic lungs (Figure 2).
- Flattened diaphragm (Figure 3).

**Figure 4.**
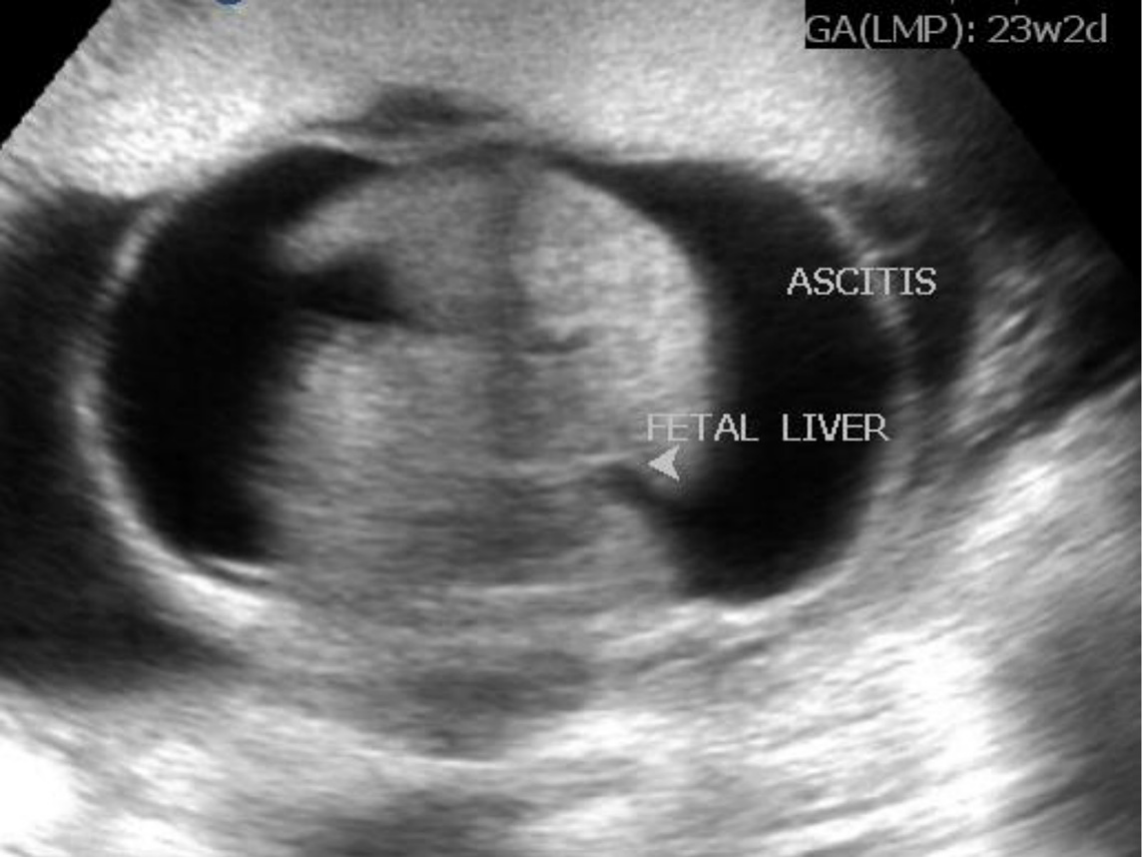
Presence of foetal ascites.

These signs were classically depicted in the foetal ultrasound scan images Figures 1–4.

## Outcome

The outcome is actually unfavourable unless EXIT procedure is considered. In this case, medical termination of pregnancy was done after prompt ultrasound diagnosis with the opinion of obstetricians.

## Discussion

Tracheal atresia is a very rare congenital malformation which takes place by deficient recanalization of the upper airways around the 10th week of gestation resulting in a clinical spectrum defined as CHAOS.^1^

Normal physiology of foetal lung involves absorption of secretions from lung parenchyma through the tracheobronchial tree. However, if there is alteration in physiological clearance of this fluid through tracheobronchial tree by any mechanical obstruction, it results in rise of intratracheal pressure. Persistent rise in intratracheal pressure leads to increased volume of lungs thereby causing compression of heart and mediastinal structures. Such compression causes cardiovascular dysfunction and reduced venous return to the right side of the heart. This leads to clinical manifestations like foetal ascites and anasarca. The enlarged lungs also cause flattening of the diaphragm and in extreme severity, inversion of the diaphragm.^2^ Tracheal or laryngeal atresia are the main causes of CHAOS. However, rare causes may be laryngeal agenesis, laryngeal webs /cysts and subglottic stenosis.^1,4^

The ultrasound evaluation plays a key role in antenatal diagnosis of CHAOS. Typical ultrasound features are bilateral boggy hyperechoic lungs, compressed and centrally repositioned heart, flattened/inverted diaphragm and ascites.^3^ There may be associated polyhydramnios or oligohydramnios depending on the type of alteration in physiological mechanism of foetal swallowing.^2^ The gestational age at the time of diagnosis may affect the amniotic fluid quantity. Polyhydramnios may not be present owing to the examination in the early 2nd trimester in most of the cases.

All the structural sonographic findings can also be recognized on MRI. The ultrasound evaluation is first-line diagnostic imaging tool owing to low cost and widespread use. However, especially if any foetal surgical intervention is planned, MR imaging can be used additionally by following the dilated airway up to the level of obstruction, as it is more effective for detecting the exact level of obstruction.^5^

CHAOS is most often misdiagnosed as bilateral congenital cystic adenomatoid malformation (CCAM).^1^ CCAM (especially Type III) and upper airway obstruction secondary to intrinsic causes such as tracheal or laryngeal atresia or stenosis and tracheal webs similarly have bilateral uniform hyperechogenic appearance of the foetal lungs on sonographic examination.^6^ In order to make a differentiation between CHAOS and CCAM Type III, the obstruction site with distal airway dilatation (present in CHAOS) and the systemic arterial supply (present in CCAM Type III) must be clearly seen.

 CHAOS should also be differentiated from extrinsic causes of tracheolaryngeal obstruction. Some of these extrinsic causes are lymphatic malformation, cervical teratoma and vascular rings like double aortic arch.^1^ (Table 1).

**Table 1.** Differential Diagnosis of CHOAS.

Anomaly	Chest involvement	Associated early ascites	Bilateral flat/inverted diaphragm	Type of lesion in chest
CHAOS	Bilateral	Present	Present	Solid
Type III CCAM	Unilateral more common	Absent	Absent	Appear as solid lesions though microcysts present
Lymphatic malformation	Unilateral more common	Rare	Rare	Cystic
Mediastinal teratoma	Unilateral more common	Rare	Rare	Solid-cystic
Double aortic arch	Bilateral	Rare	Rare	Solid-cystic

CHAOS is mostly sporadic, and the exact incidence is not known.^7^ *In utero* death of the affected cases; being a part of some genetic syndromes; better detection rate of the anomalies by the means of technical improvement of imaging tools and data only from isolated cases instead of studies consisting of large series may explain this indefinable incidence. The most common associated genetic disorder with CHAOS is Fraser’s syndrome which is inherited by autosomal recessive form and characterized by urogenital defects, laryngeal atresia, syndactyly and cryptophthalmos.^2^ CHAOS may be also a part of Cri-du-Chat syndrome, short-rib polydactyly syndrome and velocardiofacial syndrome.^8^ Apart from this, a case of CHAOS with autosomal dominant inheritance of the father and his two affected children was reported.^7^ The point to take into consideration is the necessity of a detailed evaluation of all CHAOS suspected cases due to possibility of coexistence of any genetic syndrome and significant implications of inheritance for future pregnancies.^8^

In the past, CHAOS was thought to be equivalent to a certain foetal death. However, nowadays, especially if CHAOS due to incomplete obstruction is diagnosed in the late 2nd or in the 3rd trimester and if severe hydrops has not occurred yet, the EXIT procedure (*ex utero* intrapartum treatment) can be offered. The common objective of the procedure is to settle an intact airway for the baby before the fetomaternal circulation is stopped.^9^

There are few cases with spontaneous antenatal improvements. This progress may be owing to to spontaneous perforation or tracheoesophageal fistula with the refining of the obstructed fluid leading to decrease in the pressure of airways and reversal of the process.^10^

As a result, CHAOS is a rare and fatal cause of congenital airway obstruction if unrecognized during prenatal period. Antenatal sonographic imaging shows typical findings which can lead to a diagnosis. MRI is superior to sonography in demonstrating the level of obstruction and in assisting in the differential diagnosis by excluding extrinsic causes of obstruction. This is important especially if any foetal intervention is considered.

## Learning points

1. Deviations from normal foetal anatomy should be observed very carefully to understand pathophysiology of the disease process.
2. Good quality of imaging which depicts sagittal, parasagittal and sectional anatomy of fetus helps in improving the diagnostic accuracy. This case report emphasises the benefits of good documentation.

## Consent 

Written informed consent for the case to be published (including images, case history and data) was obtained from the patient(s) for publication of this case report, including accompanying images.
